# Ensemble Learning of QTL Models Improves Prediction of Complex Traits

**DOI:** 10.1534/g3.115.021121

**Published:** 2015-08-13

**Authors:** Yang Bian, James B. Holland

**Affiliations:** * Department of Crop Science, North Carolina State University, Raleigh, North Carolina 27695; † U.S. Department of Agriculture, Agricultural Research Service, Plant Science Research Unit, Raleigh, North Carolina 27695

**Keywords:** quantitative trait loci, thinning and aggregating, ensemble modeling, *Zea mays*

## Abstract

Quantitative trait locus (QTL) models can provide useful insights into trait genetic architecture because of their straightforward interpretability but are less useful for genetic prediction because of the difficulty in including the effects of numerous small effect loci without overfitting. Tight linkage between markers introduces near collinearity among marker genotypes, complicating the detection of QTL and estimation of QTL effects in linkage mapping, and this problem is exacerbated by very high density linkage maps. Here we developed a thinning and aggregating (TAGGING) method as a new ensemble learning approach to QTL mapping. TAGGING reduces collinearity problems by thinning dense linkage maps, maintains aspects of marker selection that characterize standard QTL mapping, and by ensembling, incorporates information from many more markers-trait associations than traditional QTL mapping. The objective of TAGGING was to improve prediction power compared with QTL mapping while also providing more specific insights into genetic architecture than genome-wide prediction models. TAGGING was compared with standard QTL mapping using cross validation of empirical data from the maize (*Zea mays* L.) nested association mapping population. TAGGING-assisted QTL mapping substantially improved prediction ability for both biparental and multifamily populations by reducing both the variance and bias in prediction. Furthermore, an ensemble model combining predictions from TAGGING-assisted QTL and infinitesimal models improved prediction abilities over the component models, indicating some complementarity between model assumptions and suggesting that some trait genetic architectures involve a mixture of a few major QTL and polygenic effects.

Massive numbers of molecular markers can now be readily provided by next-generation sequencing and high-throughput genotyping (Davey *et al.* 2011; Elshire *et al.* 2011; Bian *et al.* 2014a; Glaubitz *et al.* 2014). High-density marker maps (with marker intervals smaller than 1 cM in mapping populations) have been available to an increasing number of plant species, such as in *Arabidopsis thaliana* (Kover *et al.* 2009; Huang *et al.* 2011), maize (Swarts *et al.* 2014), rice (Yu *et al.* 2011), and sorghum (Zou *et al.* 2012). Unfortunately, the dramatic increase of marker availability may adversely affect quantitative trait locus (QTL) mapping because collinearity among markers may complicate the detection and estimation of QTL. Statistical tests to declare a QTL or make QTL inference are conditional on other QTL in multi-QTL models (Zeng 1994). The value of a test statistic depends on other QTL in the model if they share some proportion of genetic variation. Strong covariance between tightly linked markers may increase the risk of selecting collinear markers, bias the QTL estimates, and possibly overfit the predictive model, especially when a relaxed selection threshold is applied (Bian *et al.* 2014b; Ogut *et al.* 2015).

Ensemble learning is an alternative approach that could improve QTL-based prediction ability. Ensemble learning involves estimating multiple “learner” models on a training data set and predicting unseen observations by a vote or weighted average among the multiple learners (Dietterich 2000). For example, suppose one has available a vector of some input variables, **x**, associated with a numerical outcome vector **y**. The estimation process involves finding a function of *F(x)* that maps values in the space of **x** to values in the corresponding space of **y**. *F(x)* can be estimated in various ways, one of which is by ensemble learning. The generic ensemble estimator takes the form: $\hat{F}\left(x\right)=h_{s}({\left\{f_{s}(x)\right\}}_{1}^{S})$, where *S* is the number of ensemble members (or “base learners”), the base learners ${\left\{f_{s}\right\}}_{1}^{S}\;$are functions of x derived from the training data, and $h_{s}\left(\cdot \right)$ is an ensemble learning function. As one example, the ensemble learner can be estimated simply by model averaging the base learners, ${\left\{f_{s}(x)\right\}}_{1}^{S}$. The base learners can be trained based on random samples from the original data set or a subset of **x**. Common ensemble methods include bootstrap aggregation of predictions (bagging) and random forests, and several features characterize them, such as loss function, ensemble dispersions, and memory of baser learners (Hastie *et al.* 2009). Bagging of regression models is trained by paralleling base regressions with no influence (zero memory) among them and minimizing squared-error loss function on bootstrap samples (Breiman 1996a). If the bootstrap estimation is roughly correct, then aggregating would reduce variance without increasing bias. Random forests (Breiman 2001) increase ensemble dispersion over bagging by additionally using a randomly chosen subset of the predictors rather than using all of them, and this method typically solves for paralleled decision or regression trees (nonlinear base learners). The ensembles of base learners produced by bagging and random forests can be conducted in a parallel manner, meaning that each individual learner is trained independent of the results from others.

Here, we developed a new ensemble learning approach used in QTL analyses, referred to as a thinning and aggregating (TAGGING) method. We thinned linkage map marker sets into submaps with equally reduced intermarker density, built QTL mapping models upon the thinned marker sets as based learners in parallel, and aggregated by averaging the predictions from the base learners to predict the test data (Figure 1). The TAGGING method shares some aspects of model averaging with bagging and random forest, as they all generate multiple predicted values and aggregate prediction by averaging. In contrast to random forests and bagging, however, TAGGING uses stratified sampling of the linkage map to create disjoint marker sets to generate independent discrete QTL models upon the unchanged set of observed mapping lines so that prediction variance is expected to decay faster and bias is not affected by bootstrapping samples. By comparison, bagging uses the original marker set to build prediction models on bootstrapped samples of lines, and random forests uses randomly sampled linkage markers and bootstrapped samples of lines.

**Figure 1:**
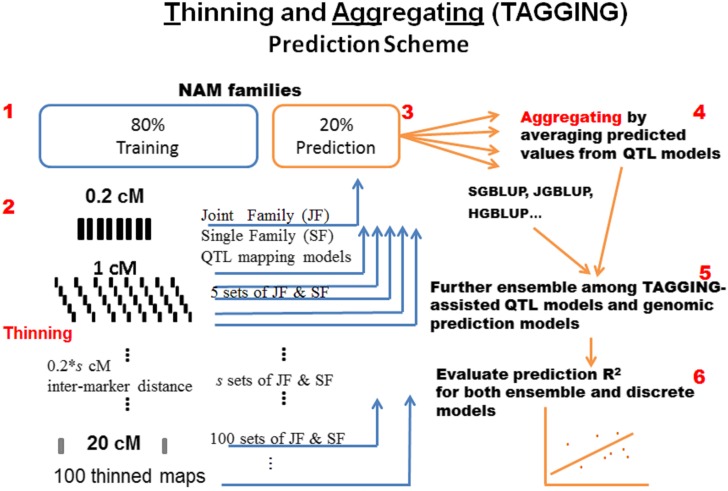
Data sampling, map thinning, model building, and ensemble schemes. The cross validations were repeated 10 times for Figure 2, Figure 3, and Figure 4 and 50 times for Figure 5. Step 1: Take a stratified random sample 80% of recombinant inbred lines of each nested association mapping family to use as a training set and 20% as test set. Step 2: Thin the original 0.2-cM resolution map into *s* sets of reduced maps with intermarker distance 0.2*s cM, and calibrate joint-family (JF) and single-family (SF) models as base learners on the same training sets with various combinations of reduced maps and *p* thresholds. Step 3: Independently predict the same test sets with the parameter estimates obtaining from training data. Step 4: Generate ensemble predictions for ensemble single-family (ESF) and ensemble joint-family (EJF) models by taking arithmetic means. Step 5: Generate ensemble learning and prediction of QTL-based and GP models. Step 6: Evaluate prediction *R^2^* for all models, including individual quantitative trait locus (QTL) models, thinning and aggregating (TAGGING)-assisted QTL models, ensembles of QTL and genomic best linear unbiased prediction models, as well as subbagging models (see text for details).

QTL mapping has proven useful for detecting major QTL with relatively large effects but may lack power in accurately modeling small QTL effects or polygenic background effects (Heffner *et al.* 2009). QTL models typically underestimate the number and overestimate the effects of QTL, reducing the accuracy of QTL-based predictions (Beavis 1998; Schön *et al.* 2004). Predictability and interpretability are two competing goals and usually compromise each other for predictive machines. The parameters in some genomic prediction (GP) models are not easily interpretable in terms of genetic theory, and this problem is compounded in nonparametric models (Gianola and Van Kaam 2008; Crossa *et al.* 2010). Such models may serve as good prediction machines but may provide no insights into the locations of important QTL or their gene actions. The TAGGING method presented here is a compromise method that aims to improve the prediction ability of QTL models while maintaining some of their interpretability in terms of QTL locations and effect sizes. Furthermore, the ensembling concept is fully general, so predictions can be obtained by ensembles of models that capture distinct components of the true genetic architecture, for example, ensembles of QTL models that are best at identifying genes with larger effects and polygenic models that capture the “background” effects of many genes of small effect distributed throughout the genome. Models that can account for two hypotheses may better approximate the true biological mechanism better than either of the two models individually.

In this study, two QTL mapping models, joint-family (JF) linkage analysis for multifamily mapping populations and single-family (SF) QTL analysis for biparental mapping populations (Ogut *et al.* 2015), were tested within the TAGGING framework for maize complex traits. The new ensemble QTL models were examined for the number of QTL detected and proportion of genotypic variance explained by the detected QTL. We expect that for a high-bias base learner (*e.g.*, QTL models based on sparse linkage maps), subset aggregation ensures model flexibility and therefore protects against high biasness in the ensemble predictions. For high-variance base learners (*e.g.*, models in which QTL are included at low selection stringency with dense maps), ensembling might provide a reduction in variance of predictions.

The objectives of this study were to: (1) develop a thinning and aggregating (TAGGING) method as a new ensemble learning approach for QTL analysis; (2) compare prediction abilities of TAGGING, QTL models, and standard GP genomic best linear unbiased prediction (GBLUP) models; and (3) test whether useful complementarity occurs between QTL-based and polygenic models in their ensemble learning for GP. We tested these models using a cross-validation strategy on data for three complex traits previously reported for the maize nested association mapping population (Buckler *et al.* 2009; McMullen *et al.* 2009). At all QTL selection stringency thresholds and thinning intensities we tested, the TAGGING strategy resulted in a better prediction result than conventional QTL methods, implying the robustness of this new method. Results indicate that TAGGING provides information on the position and effect sizes of QTL (including their precision) while improving prediction ability compared with traditional QTL mapping procedures. With very high density linkage maps becoming increasingly available, we expect the TAGGING method will find use in genetic investigations and genetic prediction.

## Materials and Methods

### Plant phenotypes and genotypes

The maize nested association mapping (NAM) population comprises a set of ∼5000 recombinant inbred lines (RILs) derived from crosses between a reference parent, inbred line B73, and 25 other diverse founder inbred lines of maize (McMullen *et al.* 2009). Three complex traits, resistance to southern leaf blight (SLB), plant height (PHT), and days to anthesis (DA), were previously studied for genetic architectures, and the predicted mean values for NAM RILs across multiple environments were previously reported (Buckler *et al.* 2009; Kump *et al.* 2011; Yang *et al.* 2013; Bian *et al.* 2014b; Peiffer *et al.* 2014) (Supporting Information, File S1, File S2, File S3, File S4, File S5, and File S6). A second-generation NAM linkage map consisting of 7386 imputed pseudomarkers with a uniform 0.2-cM intermarker distance generated from genotyping-by-sequencing followed by full-sib family haplotype imputation (Swarts *et al.* 2014) was used for linkage analysis.

Realized genomic relationship matrices (*G* matrices) were developed for each NAM family separately using the linkage map markers based on the first method described in (VanRaden 2008), which track within-family identity-by-descent (IBD) for genome segments (Table 1). The linkage map marker scores do not reflect IBD between families, however, so a separate relationship matrix for the whole NAM panel developed from the maize HapMap v1 of 1.6 million single-nucleotide polymorphisms (SNPs) (Peiffer *et al.* 2014) also was used here (Table 1 and File S7). This *G* matrix measured the pairwise relationships among all lines with reference to the hypothetical population that would result from intermating all of the mapping population F1’s. To maintain identical numbers of markers for both the within-family relationship matrices and the matrix for the whole panel, we extracted 7386 HapMap v2 markers polymorphic in NAM and closest to the 7386 linkage marker in terms of the AGP v2 coordinates (File S8, File S9, and File S10) to construct an identity-in-state (IIS) *G* matrix for the whole NAM panel. A total of 4354, 4359, and 4359 RILs with both genotypic and phenotypic data were available for analyzing SLB, PHT, and DA, respectively.

**Table 1:** Description of prediction models compared

Type	Model	Model Description	Genotypic Input	Analysis
QTL model	JF/EJF	(Ensemble) joint linkage analysis	subset(s) of linkage marker genotypes in one consensus map	(Ensemble of) joint multiple-family linkage analysis
	SF/ESF	(Ensemble) single family analysis	subset(s) of linkage marker genotypes for each of 25 families	(Ensemble of) SF analysis
	EJF+ESF	Ensemble of EJF and ESF models	As above	Average prediction of the two above
Genomic prediction model	SGBLUP	linkage map-based GBLUP model (within-family IBD)	25 ***G*** matrices from 25 sets of linkage marker genotypes	SF GP one family at a time
	JGBLUP	Allele calling-based GBLUP model (cross-family IIS)	One ***G*** matrix based on the actual genotypes most adjacent to linkage marker positions	JF GP
	HGBLUP	Allele calling-based GBLUP model (cross-family IIS)	One ***G*** matrix based on 1.6 M HapMap v1 SNPs	JF GP

QTL, quantitative trait locus; JF, joint family; EJF, ensemble joint family; SF, single family; ESF, ensemble single family; SGBLUP, single family genomic best linear unbiased prediction; JGBLUP, joint family genomic best linear unbiased prediction; HGBLUP, HapMap v-1–based genomic best linear unbiased prediction; SNP, single-nucleotide polymorphism.

### Prediction accuracy calculation

Prediction abilities of models were evaluated both for the whole NAM panel and for within each of the 25 biparental mapping population using a cross-validation procedure. Training sets were created by randomly sampling 80% of RILs from each of the 25 NAM families. The remaining lines constituted the validation sample for the SF and JF QTL models created using the training data set. Within-family predictions were evaluated, and prediction abilities were measured as the proportion of total trait variance explained by the model (*R*^2^) in the validation set. The *R*^2^ was used to enable direct comparison to heritabilities and was estimated by averaging squared Pearson’s correlations (*r^2^*) between the observed and predicted BLUP values. We converted the value of *R^2^* to have a negative sign for a few cases of negative correlations between predicted and observed line values.

### QTL models

Multiple linear regression models were first fit within each family independently (SF models; Table 1). For a given linkage maps *s*, the SF model for family *f* is:

$$\;Y^{f}=1\mu_{s}^{f}+\;X_{s}^{f}\beta_{s}^{f}+\varepsilon_{s}^{f},$$

where $Y^{f}$ is a vector of *N^f^* length referring to the trait phenotypic values in a given family, ***1*** is an *N^f^* × 1 intercept vector of 1’s, $\mu_{s}^{f}$ is the intercept, $X_{s}^{f}$ is a *N^f^* × $K_{s}^{f}$ matrix of marker genotypes, $\beta_{s}^{f}$ is a $K_{s}^{f}$ × 1 column vector of the additive effects relative to B73, $K_{s}^{f}$ is the number of significant loci in stepwise selection, and $\varepsilon_{s}^{f}$ is a *N^f^* × 1 column vector of errors. The stepwise incorporation and exclusion of markers were based on predefined *alpha* threshold (*p*). After including as many markers as possible based on their *P*-values, the model was then reduced by split-sample cross-validation. Specifically, the model selection step with minimum prediction error variance was chosen in five-fold split-sample cross-validation within the training set, using the ‘choose = CV cvMethod = split(5)’ option in SAS Proc GLMSelect (SAS Institute 2013). The RIL phenotype values were predicted from the linear model.

JF linkage models were trained in all 25 families of NAM populations. For a given linkage maps *s*, JF analysis for the multifamily connected populations can be described as:

$$Y=A\mu_{s}+\;\overset{K_{s}}{\underset{i=1}{\sum }}X_{\mathrm{si}}\beta_{\mathrm{si}}+\varepsilon_{s},$$

where ***Y*** is a vector of *N* length referring to the trait phenotypic values for all RILs, **A** is an *N* × *P* incidence matrix relating RILs to their corresponding family, $\mu_{s}$ is a *P* × 1 column vector for the family effects, $X_{\mathrm{si}}$ is a *N* × *P* matrix relating each RIL’s genotype at locus *i* to its corresponding family-specific allele effect, $\beta_{\mathrm{si}}$ is a *P* × 1 column vector of the family-specific additive effects associated with locus *i*, $K_{s}$ is the number of significant loci in stepwise selection based on pre-defined *p*, $\varepsilon_{s}$ is a *N* × 1 column vector of errors. The prediction ability was then evaluated for each family separately to make a comparable comparison with biparental scenarios.

### TAGGING method for QTL analyses

The TAGGING QTL analysis method can be summarized as thinning of dense marker maps into a set of disjoint maps of lower density and then aggregating predictions from paralleled QTL models on the same training data sets (Figure 1). The method begins by conducting a stratified sampling of the markers on the linkage map, which is simplified in the case of the maize NAM linkage map which has a uniform density of 0.2 cM between each pair of adjacent linked markers. The original map is thinned into *s* disjoint sets of markers, maintaining the linkage map position information for the markers, starting with the first marker on the first linkage group. This procedure was then repeated by starting selection at the second marker to create a new sample map and continuing to initiate selections at subsequent markers until the *s*^th^ marker. The result is *s* disjoint maps, each with s*0.2 cM distance between adjacent markers. Thinning is expected to reduce the extent of covariance and collinearity that otherwise occur in base QTL analyses with the dense maps and/or relaxed selection stringencies.

JF and SF models for multifamily and biparental mapping populations were fit using each thinned map separately, and phenotype values for the validation set lines were predicted separately for each reduced map (File S11 and File S12). The two types of QTL base learners were constructed with each subset of markers for the same training samples to estimate the regression coefficients, and then the *s* sets of SF and JF predicted values for the test sets were aggregated to form the ensemble prediction (Figure 1). The ensembles of QTL models proposed here were named “ensemble joint-family linkage” (EJF) analyses for multifamily mapping populations and “ensemble single-family” (ESF) analyses for biparental mapping populations, respectively. The ensemble learner $\hat{F}\left(x\right)$ is formed as some linear combination of predictions of each base learner: $\hat{F}\left(x\right)=a_{0}+\;\overset{S}{\underset{s=1}{\sum }}a_{s}f_{s}(x)$, with ${\left\{a_{s}\right\}}_{0}^{S}$ being the coefficients for ${\left\{f_{s}(x)\right\}}_{1}^{S}$. For both EJF and ESF modeling, the coefficients were set to $a_{0}=0$ and ${\left\{a_{s}=1/s\right\}}_{1}^{S}$. Essentially, we averaged predictions over *s* map subsets for each training set, and the ensemble predictions were an arithmetic mean of those base learners.

To estimate precision of QTL localization, the frequencies of QTL positions detected in JF and EJF across the resampled training sets were summarized to elucidate the QTL architectures for the three complex traits. Resample model inclusion probability (RMIP) (Valdar *et al.* 2009) was computed to measure the power of detection for the trait-marker associations across NAM panel. The RMIP was calculated for each marker as the proportion of data samples in which the marker was tested and selected in the model of interest at the given selection *p*.

To compare the efficiency of the TAGGING ensemble learning method with existing ones, we implemented subsample aggregating (subagging) of both JF and SF analyses using the same base learning algothrims as in TAGGING. Subagging is a sobriquet for subsample aggregating where simple random sampling is used instead of aggregation of bootstrapped samples implemented by bagging. The basic difference between TAGGING and subagging is that TAGGING averages over subsamples of markers on a fixed set of RILs, while subagging (subsample aggregating) averages over samples of RILs for a fixed set of markers. Subagging instead of bagging was used here because subbaging is more efficient in computation, because of reduction in sample size compared with bagging, and it avoids collinearity problems induced or aggravated from the increased relatedness among bootstrapped samples, which is a concern with the dense linkage map used here. We formed 10 simple random sampling subsamples with sample size equal to 80% of each family from each original training set. We implemented subbagging of both SF and JF analyses upon the same training and test sets as used by all other analyses.

SF and ESF models were constructed using selection thresholds ranging from *P* = 1e-4 to *P* = 0.05, and *P* = 1e-5 to 0.01 for JF and EJF. Map resolutions used ranged from 0.2 to 20 cM intermarker distances for both. Because *P* = 1e-05 was too stringent for SF, and *P* = 0.01 or greater were too relaxed for JF using 0.2-cM density map (Ogut *et al.* 2015), we did not include them in this study.

### Prediction error decomposition

The cross-validation experiments allow us to evaluate the mean squared prediction error and to decompose this into terms due to the bias and variance in prediction. Suppose there is some underlying true function f(x) estimated from ensemble or discrete QTL models on the training set T by h(x,T). Given a new data point in test set $x^{*},\;\;y^{*}=\;f\left(x^{*}\right)+\;\varepsilon$ where *ε* is normally distributed with zero mean and variance $\sigma^{2}$, a function of 1 − heritability. The expected pointwise prediction error can be decomposed in a familiar form:

$$E_{T}\left[{\left(y^{*}-h\left(x,T\right)\right)}^{2}\right]=\;E_{T}\left[{\left(y^{*}-E_{T}[h\left(x,T\right)]\right)}^{2}\right]+\;E_{T}\left[{\left(\;h\left(x,T\right)-E_{T}[h\left(x,T\right)]\right)}^{2}\right],\;$$ (1)

where $E_{T}$ denotes expectation over resampled training sets drawn from the same distribution to estimate $y^{*}$. Whereas the second part of the right hand side of (1) is prediction variance, the first part of the right hand side of (1) is the sum of bias^2^ and irreducible error variance and decomposed as:

$$\begin{array}{l} E_{T}\left[{\left(y^{*}-E_{T}[h\left(x,T\right)]\right)}^{2}\right]=E_{T}\left[{\left(f\left(x^{*}\right)-E_{T}\left[h\left(x,T\right)\right]\right)}^{2}\right]+\;E_{T}\left[{\left(y^{*}-\;f\left(x^{*}\right)\right)}^{2}\right]\;\;\;\;\;\;\;\;\;\;\;\;\;\;\\ =\;E_{T}\left[{\left(f\left(x^{*}\right)-E_{T}\left[h\left(x,T\right)\right]\right)}^{2}\right]+\;\sigma^{2}\;,\; \end{array}$$ (2)

The TAGGING method estimated h(x,T) by averaging over *s* predictions from *s* sets of thinned marker maps based on a training set T used and estimated $E_{T}\left[h\left(x,T\right)\right]\;$by averaging over all test sets that contain the data point in question. Finally, averaging over all points predicted throughout all test sets for their bias and variance gives the more accurate estimates. We refer to $E_{T}\left[{\left(y^{*}-E_{T}[h\left(x,T\right)]\right)}^{2}\right]$ in (1) as “bias^2^” in our results, because the true underlying genetic mechanism is unknown and thus the irreducible error variance could not be disentangled from the bias^2^ in (2). The bias^2^ and variance in prediction were compared between single and TAGGING-based models all of which used the same 7386 linkage marker genotypes. In addition, a few predicted values representing the most severe outliers in prediction ability were excluded from prediction error calculation in order to guard against artifactual inflation of the prediction error variance simply due to high collinearity that occasionally occurred in few cross-validations. The cutoff for excluding an outlier was the mean predicted value ± 100 times the standard error of the mean.

### GBLUP models using IIS and IBD genotypes

The *G* matrix derived from 1.6 million SNPs in HapMap v1 was used to create a GBLUP model for the entire NAM population HapMap v1–based genomic best linear unbiased prediction (HGBLUP) models, Table 1) in the same cross validation setup as in QTL analyses. single-family genomic best linear unbiased prediction (SGBLUP; Table 1) model were developed for each NAM biparental mapping family using the 25 disjoint *G* matrices developed from the original linkage map of 7386 markers. We also extracted the same number of allele calls as close as possible to the physical positions of the 7386 linkage map pseudomarkers, and with the IIS *G* matrix across whole NAM panel derived from that, we conducted the joint-family genomic best linear unbiased prediction (JGBLUP) models (Table 1) to compare the efficiencies of using the same amount of genotypic information in different models.

### Ensemble learning of TAGGING-assisted QTL models and GP models

To capture the complementary strengths of SF and JF analyses, we averaged predictions from EJF and ESF and referred to this ensemble as the EJF + ESF model. All pairs of QTL and GP models using the same number of 7386 loci were then combined in ensemble learning in a linear fashion. We explored ensemble learning by averaging predictions from either EJF, ESF, or EJF + ESF QTL models with predictions from SGBLUP or JGBLUP models to test the hypothesis that mixture of models with contrasting genetic assumptions can improve genetic prediction by better mimicking genetic architectures (File S13). For simplicity, the coefficients in ensembles of two component models were set to 0.5. Finally, we attempted ensemble learning that combined QTL and GBLUP models derived from both 7386 marker genotypes and 1.6 M HapMap1 SNP genotypes. We evaluated the importance of the four participating models to their ensemble prediction *R^2^*, EJF + ESF, SGBLUP, JGBLUP, and HGBLUP, by analysis of variance in a 2^4^ – 1 factorial experiment, in which 15 possible combinations of the four models’ presence or absence were included.

### Pseudooptimal ensemble coefficients

We tested whether additional tweaking of the coefficients of the base leaners in the ensemble model can improve the ensemble prediction. The base learners can be considered as input variables to a multiple regression model aimed at predicting the unknown trait values. Here we introduced pseudooptimal coefficients for giving relative weights to each component model, which we estimated from using cross-validation with respect to the true phenotypic data of test sets to identify a fixed set of ensemble coefficients to minimize the least-square errors for the test data. The ensemble coefficients were tuned using the Nelder–Mead optimization technique (Nelder and Mead 1965), in which average prediction *R^2^* values were dynamically recorded for tested sets until they (as objective functions) converged to a plateau. The sum of coefficients was scaled to one, under nonnegativity constraints, as recommended by Breiman (1996b). The pseudooptimal accuracies should be considered as an idealistic upper limit and not likely to occur in a real prediction scenario, as those predictions were obtained using the phenotypic values in test sets to optimize the ensemble coefficients. Finally, two sets of natural coefficients were attempted in ensembles of the aforementioned three component models, EJF, ESF, and SGBLUP: equal weights of 1/3 for each, and equal weights between QTL-based models (0.25 for EJF and ESF) and SGBLUP models (0.5).

### Data availability

Seeds of NAM lines have been deposited at USDA Maize Genetics Cooperation Stock Center (http://maizecoop.cropsci.uiuc.edu/nam-rils.php). File S1, File S2, File S3, File S4, File S5, File S6, File S7, File S8, File S9, File S10, File S11, File S12, and File S13 are available for download at http://downloads.figshare.com/article/public/1517823.

File S1 (NAMSLB.RData) contains mean values for Southern leaf blight disease scores and linkage map marker scores for NAM RILs formatted as an R data object. File S2 (SLB_genopheno.sas7bdat) contains mean values for Southern leaf blight disease scores and linkage map marker scores for NAM RILs formatted as a SAS data set. File S3 (NAMPHT.RData) contains mean values for plant height and linkage map marker scores for NAM RILs formatted as an R data object. File S4 (PHT_genopheno.sas7bdat) contains mean values for plant height and linkage map marker scores for NAM RILs formatted as a SAS data set. File S5 (NAMDA.RData) contains mean values for days to anthesis and linkage map marker scores for NAM RILs formatted as an R data object. File S6 (DA_genopheno.sas7bdat) contains mean values for days to anthesis and linkage map marker scores for NAM RILs formatted as a SAS data set. File S7 (relMat.RData) contains the realized additive genomic relationship matrix for NAM lines based on 1.6M HapMap I markers (courtesy of Dr. Jason Peiffer). File S8 (NAMSLB_IIS.RData) contains mean values for Southern leaf blight disease scores and Identity In State (IIS) calls for HapMap markers closest to NAM linkage map markers for NAM RILs formatted as an R data object. File S9 (NAMPHT_IIS.RData) contains mean values for plant height and Identity In State (IIS) calls for HapMap markers closest to NAM linkage map markers for NAM RILs formatted as an R data object. File S10 (NAMDA_IIS.RData) contains mean values for days to anthesis and Identity In State (IIS) calls for HapMap markers closest to NAM linkage map markers for NAM RILs formatted as an R data object. File S11 (ESF.sas) contains SAS code to conduct ensemble single family QTL analysis. File S12 (JSF.sas) contains SAS code to conduct ensemble joint family QTL analysis. File S13 (GBLUP and ensemble model prediction SLB.R) contains R code to ensemble GBLUP and ensemble-QTL-based predictions.

## Results

### TAGGING of QTL models improves QTL model prediction

**Figure 2:**
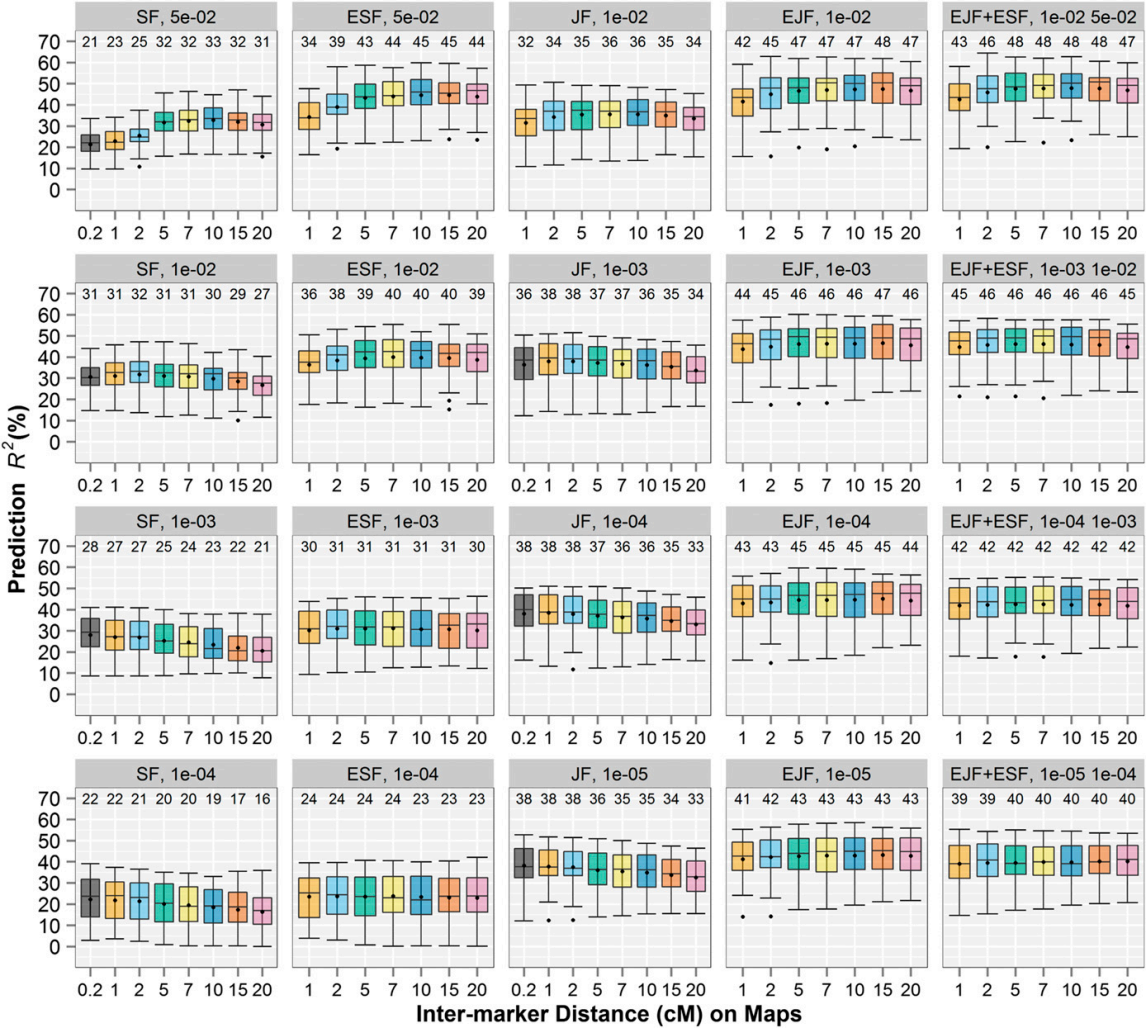
Prediction *R^2^* for resistance to southern leaf blight in biparental and multiple-family prediction, comparing joint-family (JF), single-family (SF), and the thinning and aggregating (TAGGING)-assisted quantitative trait locus (QTL) analyses using multiple map densities and under multiple selection *p*. The number at the top of and dot within each boxplot present the mean *R^2^* among 25 nested association mapping families for that boxplot. The x-axis represents the densities of linkage maps used for the JF, SF, and TAGGING-assisted QTL methods.

EJF models had substantially better prediction *R*^2^ compared with the individual JF models using either the original or reduced maps for the three traits (Figure 2, Figure S1, Figure S2, Table S1, Table S2, and Table S3). Using the thinned maps of 10 ∼15 cM intermarker distances at selection *P* = 0.01 generated the best prediction *R^2^* in EJF for SLB (*R*^2^ = 0.475), PHT (0.476), and DA (0.445); those best EJF models outperformed the best JF model substantially [1 cM maps under *P* = 0.0001 for SLB (*R^2^* = 0.385) and PHT (0.371), and 1 cM maps under *P* = 0.001 for DA (0.332)]. The improved prediction abilities of EJF over JF indicated that the genetic architecture can be better depicted by averaging multiple independent QTL models developed from disjoint subset maps as achieved by TAGGING. TAGGING of SF analyses also improved the predictions for the biparental mapping populations substantially compared with the regular SF analyses. The prediction *R*^2^ values generated by the optimal ESF models outperformed those from the best SF models by an average of 0.118, 0.123, and 0.116 across 25 families for SLB, PHT and DA, respectively (Figure 2, Figure S1, Figure S2, Table S1, Table S2, and Table S3). Among the pure linkage models (SF, JF, ESF, and EJF) and across all traits, map densities, and selection *p* thresholds tested, EJF models had the greatest prediction abilities, whereas SF models always had lowest prediction ability (Table S1, Table S2, and Table S3).

The performances of both discrete and TAGGING QTL models varied with the densities of maps and selection *p*. In general, for EJF, sparse maps and relaxed *p* were favorable in prediction, and for JF, dense maps and stringent *p* were favorable. Prediction abilities from EJF increased with decreasing *p* thresholds and decreasing map densities until the map density reduced down to 15−20 cM intermarker distances (Figure 2, Figure S1, and Figure S2). In contrast, for biparental mapping populations, moderate map densities along with relaxed *p* were advantageous in prediction using both SF and ESF analyses. In addition, using linkage maps of 1 cM or 0.2 cM density made little difference in prediction abilities for both JF and SF QTL models (Figure 2, Figure S1, and Figure S2).

The efficiency of TAGGING was compared with the previously proposed ensemble learning method subbagging, both of which were applied in the same inputs and cross validation schemes. The best prediction *R^2^* values were 0.452, 0.440, and 0.405 using subbagging of JF models under selection *P* = 0.001 and 0.396, 0.378, and 0.391 using subbagging of SF models under selection *P* = 0.01 for SLB, PHT, and DA, respectively (Table S1, Table S2, and Table S3). In all cases, the optimal prediction abilities of TAGGING-assisted QTL models were superior to the corresponding subbagging predictions (Table S1, Table S2, and Table S3).

### Bias and variance

**Figure 3:**
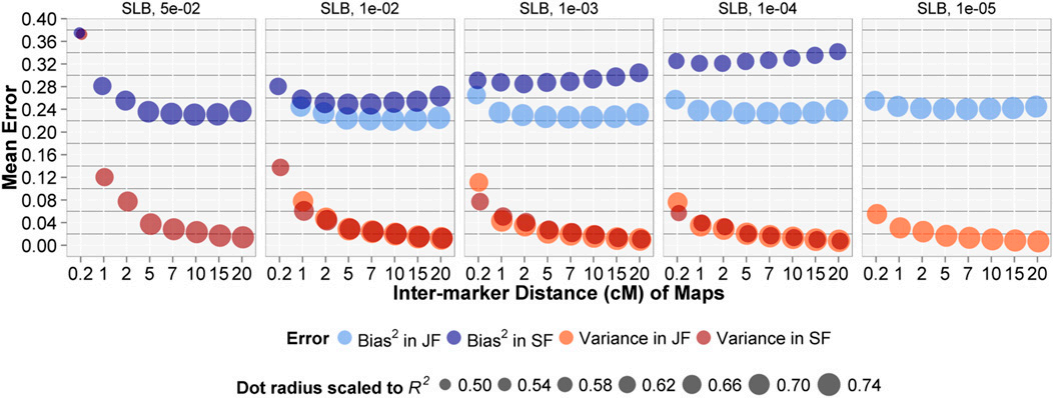
Prediction bias^2^ and variance in thinning and aggregating (TAGGING)-assisted and single quantitative trait locus (QTL) models for resistance to southern leaf blight (SLB). X-axes denote the intermarker distances for the genetic maps used: “0.2” for single joint-family (JF) or single-family (SF) models, and others for TAGGING methods. Dot radius was scaled to within-family *R^2^* calcualted based on all recombinant inbred lines in the test sets to permit comparisons of both mean error measures and mean prediction *R^2^*.

For all traits and under all selection *p* thresholds, TAGGING substantially reduced prediction variance in both JF and SF models (Figure 3 and Figure S3). Variance in prediction was basically eliminated when a large (≥25) number of thinned maps were averaged in TAGGING. TAGGING of JF models always reduced the bias compared with the single JF models, and the magnitude of bias in EJF models was roughly equal across a large range of thinning intensities, even when the map density was as low as 100 markers per map. The prediction bias in TAGGING of SF analyses can fluctuate compared with that of the individual SF models, depending on the thinned map densities (Figure 3 and Figure S3). When SF models suffered from severe collinearity at the relaxed selection stringency (*P* = 0.01), thinning reduced covariance among markers and alleviated model bias. When SF models were based on high stringency of marker selection (*P* = 1e-04), aggregating of the appropriately thinned maps (2 ∼5 cM inter-marker distances) seemed to slightly improve model fit by incorporating more predictors in the ensemble. In general, TAGGING seemed to be more protective against high bias for JF than SF models (Figure 3 and Figure S3). By examining the error compositions, we found that the prediction advantages of TAGGING of JF models over that of SF models were related to the lower level of bias before using TAGGING and more stable reduction of bias after using TAGGING (Figure 3 and Figure S3). In addition, when comparing across *P*-value thresholds, prediction bias in both TAGGING models decreased with more relaxed *P*-values because of greater model flexibility.

### Genetic architecture revealed by marker-trait associations

**Figure 4:**
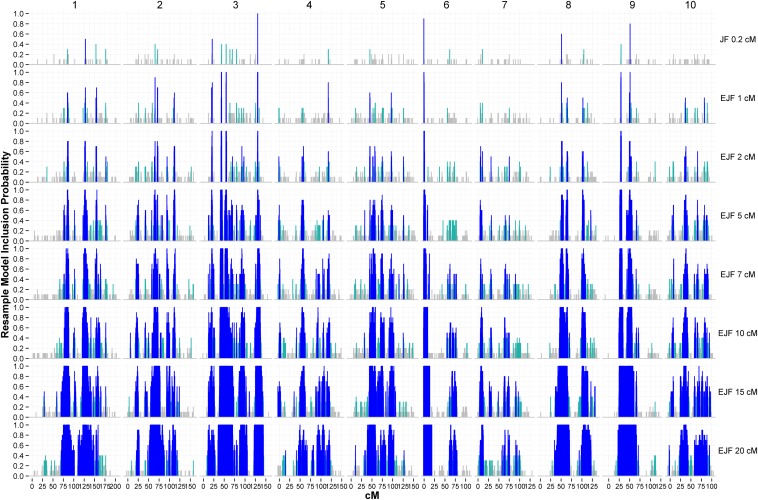
Marker-trait associations identified by joint-family (JF) and ensemble joint-family (EJF) models across 10 chromosomes, using different map densities under selection *p* at 0.001 for maize SLB resistance in nested association mapping panel. Blue, cyan, and gray peaks denote associations with resample model inclusion probability (RMIP) values greater than 0.5, 0.3−0.5, and less than 0.3, respectively. X-axes deonte the genetic positions (cM) across 10 chromosomes. RMIP values are summarized at the individual marker (every 0.2 cM) basis.

To better understand the trait QTL architectures, the probability of inclusion of a marker in a JF or EJF models was estimated across the resampled training sets (resample model inclusion probability, RMIP; Valdar *et al.* 2009). RMIP plots visualized the enrichment of marker-trait associations within particular genomic regions (Figure 4, Figure S4, and Figure S5). In general, with the decrease in the thinned map densities used in EJF (which also reflects the increase in the number of JF models combined by TAGGING), RMIP values increased substantially and the regions containing marker associations expanded (Figure 4, Figure S4, and Figure S5). As the selection *p* relaxed, the RMIP values in EJF increased and the enriched regions expanded, especially for sparse maps (Figure S6).

### GBLUP model prediction performance

The same marker data used for SF and ESF models also were used to construct within-family realized genomic relationship matrices. These relationship matrices were used to implement SF GP models (SGBLUP model) (Table 1). Average within-family prediction *R*^2^ values were 0.460, 0.465, and 0.450 for SLB, PHT, and DA, respectively, using the SGBLUP model. The JGBLUP model, which used an integrated relationship matrix calculated based on IIS information of the same number of marker genotypes that were closest to the linkage marker physical positions, generated best prediction results among EJF, ESF, SGBLUP, and JGBLUP models (Table S4). Similarly, a previous report showed that joint analyses of several half-sib families in GP models can increase prediction abilities over family-specific GP models (Lehermeier *et al.* 2014). In addition, the NAM panel was also analyzed using HGBLUP model for which the relationship matrix was calculated based on the 1.6 M HapMap v1 SNPs. HGBLUP generated the best prediction abilities of all component models (Table S4) probably because of the enormous amount of genotype information used.

### Ensemble of TAGGING-assisted QTL models and GBLUP models

The SF and JF QTL models were based on different genetic assumptions of genetic heterogeneity and allele effects, and may be considered complementary in describing QTL architectures (Ogut *et al.* 2015). To test the hypothesis that the combined QTL mapping results can improve prediction, we ensembled by model averaging to combine results from EJF and ESF. The EJF + ESF models did not noticeably improve prediction abilities over the EJF models (Figure 2, Figure S1, and Figure S2). The EJF + ESF results indicated that the selection *P* = 0.001 and 0.01 in EJF and ESF analyses resulted in the optimal prediction *R^2^* when 1 cM reduced maps were used, and *P* = 0.01 and 0.05 for 20 cM reduced maps (Figure 2, Figure S1, Figure S2, Table S1, Table S2, and Table S3). We then restricted our modeling to using those *P* thresholds for the most thinned 20-cM maps in further ensemble learning.

**Figure 5:**
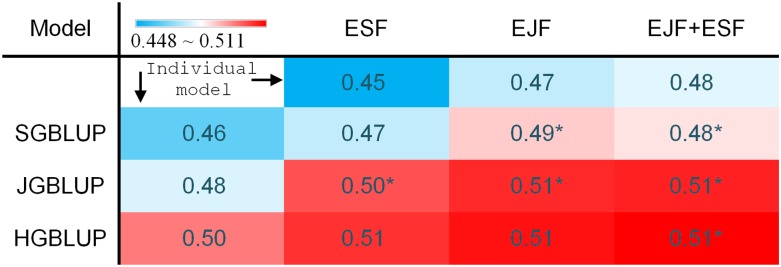
Ensemble between thinning and aggregating (TAGGING)-assisted quantitative trait locus (QTL) and genomic best linear unbiased prediction (GBLUP) models showed their complementary effects on prediction ability. The mean within-family prediction *R^2^* values for QTL and GBLUP models were shown in first row and column, and other cells show the *R^2^* values for ensembles with equal weights for the two models in the corresponding rows and columns. The thinned maps of 20-cM intermarker distances were used for the QTL models, under the best selection stringency [*P* = 0.01 for ensemble joint family (EJF); 0.05 for ensemble single family (ESF)]. *P-value < 0.0005 in the one-sided binomial tests with the null hypothesis that the ensemble model predicted the same as the corresponding row GBLUP model (≥21 of 25 families). The *R^2^* values were estimated from 50 replicates of cross validation. Standard errors of prediction ability based on variation among families ranged from 0.016 to 0.018. JGBLUP, joint-family genomic best linear unbiased prediction; HGBLUP, HapMap v1-based genomic best linear unbiased prediction.

For ensemble learning between TAGGING-assisted QTL and GBLUP models, first, we attempted pairwise combinations of the three QTL models and two GBLUP models that used the same underlying 7386 marker genotypic information. Adding EJF or EJF + ESF models substantially increased within family prediction *R^2^* and generated better prediction results for at least 21 (*P*-value < 0.0005) out of 25 NAM families, compared with their ensemble partner SGBLUP or JGBLUP models (Figure 5, Figure S7, and Table S4). Second, the best ensemble combinations of QTL and GBLUP models that used the same limited genotypic information improved prediction abilities, resulting in comparable performance relative to GBLUP models using much larger scale genotypic information. Specifically, (EJF + ESF) + JGBLUP models outperformed HGBLUP models (based on >200 times more marker information) by 0.01 ∼0.02 in within-family prediction *R^2^* for SLB and PHT (Figure 5, Figure S7, and Table S4).

The high consistency of model prediction abilities between individual component models and their ensemble models suggested that the ensemble learning performance is likely predictable. Indeed, linear models involving terms of HGBLUP and JGBLUP or (EJF + ESF)*JGBLUP explained ∼72% to ∼84% (Table S5) of the variance in within family prediction *R^2^* among all combinations of the four tested component models (EJF + ESF, SGBLUP, JGBLUP, and HGBLUP). The (EJF + ESF) + HGBLUP + JGBLUP model (based on equal weights of 1/3 for the three components) resulted in the best prediction *R^2^* (0.516 for SLB, 0.515 for PHT, and 0.512 for DA), which was 0.02 better for SLB and PHT and marginally better for DA than the HGBLUP model; it also predicted better in significantly more families than the HGBLUP model (Table S4). The magnitude of the increase in prediction *R^2^* from ensembling TAGGING-assisted QTL models and GBLUP models was small but consistent across families. The traits studied are highly multigenic, so the results may be considered as a conservative case for evaluating the utility of the additional QTL information in GP models.

### Pseudooptimal ensemble coefficients

We further searched for a fixed set of optimal coefficients that can maximize the accuracy from the EJF, ESF, and SGBLUP predictions. The prediction accuracy reached plateau after several rounds of Nelder–Mead optimizations. The resulting average within family prediction *R^2^* based on the “pseudooptimal” ensemble coefficients differed by less than 0.01 from the two sets of simple coefficients (1/4)EJF + (1/4)ESF + (1/2)GBLUP and (1/3)EJF + (1/3)ESF + (1/3)GBLUP we developed (Table S6). The results implied that the current ensemble learning to combine QTL-based and GP models was efficient in capitalizing on the model complementarities.

## Discussion

### Optimizing QTL mapping parameters

Determining the optimal prediction model for a collection of multiple biparental families, as is commonly encountered in both academic genetic studies and commercial breeding programs, is important for maximizing precision of QTL mapping and accuracy of genome-enabled prediction. Our results demonstrate that there is an interaction between map density and QTL significance threshold on model prediction performance. The optimal threshold for a given analysis can vary according to map density. In earlier studies of prediction accuracy within biparental families from QTL models, accuracy was optimal at quite relaxed *P*-value thresholds (Hospital *et al.* 1997; Bernardo 2004). Those studies were conducted by the use of linkage maps with substantially lower marker density than the current study. As genomics technology has made increasingly dense linkage maps available, however, marker colinearity becomes problematic for QTL mapping and the relaxed threshold is no longer optimal. For example, when *P* = 0.05 were adopted in SF analysis at the greatest map density, average prediction ability was 0.21, and this improved to 0.31 by increasing the selection stringency to *P* = 0.01 (the first column of Figure 2). Using appropriately chosen selection *p* reduced the propensity to overfitting or underfitting resulting from the improper marker densities. Nevertheless, an overly sparse map used in JF or SF models may fail to capture the QTL information or compromise the precision of QTL positioning because the markers are not in tight linkage to many QTL and therefore lead to omitted-variable bias (Figure S8). Our JF and SF model results suggest that a 1-cM density is appropriate for the level of linkage disequilibrium (LD) that exists within a biparental RIL family in maize. By comparison, previous research reported that marker densities increasing from 5 to 1 cM in a doubled haploid maize population improved neither the overall QTL detection power nor the proportion of genotypic variance explained by the detected QTL (Stange *et al.* 2013). Finally, our results confirmed that the optimal selection threshold also differs by QTL mapping design. SF models are more prone to underfitting (high bias) problems than JF models, and more stringent selection threshold should be used in multiparental analysis (Blanc *et al.* 2008). To conclude, it is important to consider the dynamics among map density, QTL analysis method and QTL detection stringency, the optimization is important to gain the best performance in QTL analysis based prediction (Figure 2, Figure S1, and Figure S2). Performances of both traditional and TAGGING-assisted QTL models varied with the densities of maps and selection *p*. However, the prediction ability of TAGGING models was greater than the respective traditional QTL mapping methods for all selection *p* and thinning intensities tested.

Bayesian GP models represent an important class of models to consider, but the computational difficulty of conducting on many repeated samples with the millions of markers used in this study (for the HGBLUP models) precluded their inclusion in the model comparisons in this study. In addition, Bayesian models have shown little or no advantage over GBLUP models in many plant breeding scenarios (Heslot *et al.* 2012; Guo *et al.* 2012).

### Marker-trait association and genetic architecture

The EJF models may reflect the true genetic architecture better than discrete models concerning only a few strong associations. Indeed, the greater number of marker associations contributing to ensemble predictions is one of the bases for the improved prediction ability. By using the TAGGING method, association architectures in the EJF models were equipped with the polygenic feature that can include many more markers linked to QTL, while it also involved marker selection and differential weighting of marker information. The precision of QTL mapping, however, can be compromised in ensemble mapping. For example, previous data had indicated with high confidence the presence of two linked QTL on chromosome 3 for genetic resistance to SLB (Bian *et al.* 2014b), but the blurring of QTL positions in EJF resulted in a merging of the two QTL into a single broad peak in the RMIP visualizations (Figure 4). The larger number of marker effects that are ensembled in the TAGGING method may offer a useful compromise between QTL detection and predictions. We focused on cross-validation comparisons based on real data here, but simulation studies would be required to determine the accuracy of QTL positition and effect estimates from the TAGGING method.

Ogut *et al.* (2015) suggested that JF models (which assume common QTL positions among families) and SF models (which assume independent QTL positions among families) can complement one another by capturing different aspects of the overall genetic architecture. We tested this hypothesis by ensembling predictions from the two QTL models in a single EJF + ESF model. The ESF + EJF model appeared to offer little advantage over EJF: the relatively poor predictive ability of ESF negated any advantage of complementarity between ESF and EJF models when they were ensembled. A single integrated base learner model that more flexibly fits allele effects only within families where they are significant may more effectively achieve the goal of taking advantages of both models in depiction of genetic architectures. Development of such a model is underway.

Marker-trait associations are found highly enriched in some genomic regions, as indicated by the disjoint TAGGING scans. For a particular region of interest *a priori*, the RMIP information surrounding that region indicates its importance and the resolution of QTL information. The association-enriched regions might represent probable intervals of QTL effects, that is, QTL or the cluster of QTL may reside in the multi-locus regions or in linkage with the loci in the regions. Leveraging association-enriched regions may better explain the underlying genetic architectures compared to the traditional point estimates of single QTL peaks, although further research remains needed to effectively define and set boundaries for the regions or factor in their kernel density. We suspect that the association-enriched regions might be important to reveal hidden genetic variation. Further research will be required to develop this method and test the model efficiency.

### Bias and variance in TAGGING prediction

The prediction ability of ensemble learning is usually stronger than that of a single learner. The first reason is that the training sample size might not be sufficient for determining a single best base model. In TAGGING, the reduced maps provide similar information, because they only differ by consecutive markers. The base learners should perform similarly well on searching those slightly different hypothesis spaces to fit the same training data sets. Thus, combining these learners can join the marker information that would otherwise not be obtained by searching in the original hypothesis space with a single algorithm (Rokach 2010). The second reason is that the search processes of the learning algorithms might be imperfect, especially in the “small N, large P” situations that have become common in genomics. Even if there exists a unique best set of predictors (actual functions of genes underlying the studied traits), the high dimensionality of dense linkage maps may hinder this set from being selected by efficient search algorithms. Thus, ensembles can compensate for such imperfect search processes by reducing the hypothesis space. The third reason is that, the model specification (linear models here) being tested might not contain the true target function, but ensembles can nevertheless provide better approximations to the true function than a single base learner function can.

The proposed TAGGING framework was successful at strengthening “weak learners” (two types of QTL models) by first reducing the hypothesis space and then aggregating by averaging the base models. A rule of thumb for optimizing TAGGING in QTL-based prediction is to conduct EJF based on heavily thinned maps (more sets to aggregate) under relaxed selection *p* thresholds. Thinning alleviates the collinearity within each marker set, and this allows the selection threshold to be relaxed without overfitting. In addition to thinning, aggregating across predictions from multiple models also decreases the prediction variance. For example, averaging *N* identical independent model predictions would reduce the resulting prediction variance by a factor of 1/*N*. In most ensemble learning, including TAGGING, reduction is obviously less than 1/*N* because of dependent base predictions. Thinning by stratified sampling of markers from the linkage map takes advantage of the consistent ‘spatial’ pattern of correlation among markers, such that the subset hypothesis spaces defined by thinning represented disjoint representations of the linkage map. By decomposing the prediction error variances, we showed that the contribution of variance among prediction sets was reduced to almost zero with large samples of sparser subset maps (Figure 3). Finally, the TAGGING strategy tracks the linkage structure exactly in our case of a perfectly uniform marker density. In more typical QTL mapping situations in which the markers are not evenly spaced in the linkage map, it is important to stratify maps accounting for the original marker correlations, instead of just even sampling across chromosomes. Similarly, in genome-wide association studies for which the correlation structure of marker set is always not constant, the LD between marker genotypes does not decays monotonically as their physical distances extend. Special attention needs to be paid to appropriate marker stratification schemes for these more general situations of uneven marker spacing for QTL mapping and complex LD structure for association mapping when TAGGING.

The minimum density required for TAGGING to work was around 20 cM in the thinned maps, and further thinning caused decreased prediction ability in TAGGING. Because dense maps are expected to be more easily available for many species, the applicability of TAGGING will only increase over time. Furthermore, most crop plants regularly deal with maps with density greater than one marker per 20 cM, so there is already general applicability at this time.

In addition to reducing the variance by model averaging, another motivation of TAGGING was to alleviate bias in prediction. First, a large collection of disjoint predictors can be considered and weighted when ensembling them, and therefore more genotypic information (Figure 4, Figure S4, and Figure S5) contributes to the ensemble prediction, resulting in reduced bias in prediction. Moreover, when a few dominating predictors consistently perform better than their correlated competitors (for example, markers within the same LD block), they will tend to be selected in prediction models at the expense of those weaker competing predictors, some of which may provide information about local features of the data. TAGGING thins the map into equally spaced disjoint maps, providing more opportunity for predictors to be considered without competition from the dominant predictors, possibly increasing the chance that weak local features will be represented by some of the base learners.

We expect that bootstrapping of data samples in addition to TAGGING to generate even greater numbers of base learners will not result in a substantial decrease in the prediction error, as the variance is already nearly eliminated by TAGGING, whereas bias may increase due to the use of smaller effective training samples for each base learner. As observed here (Figure 3 and Figure S3) and found in many other ensemble studies, the dominating error source turns out to be the bias^2^ and irreducible error (Bauer and Kohavi 1999), which suggests necessity of bias reduction in ensemble learning. A better approach may be to implement TAGGING upon greater variable (lower bias) base learners. Moreover, bias corrected estimators (Efron 1987) can be further incorporated into TAGGING, which may help reduce the prediction bias due to finite-sample bias in base learner estimators (Horowitz 2001; Zhang and Lu 2012). Similar to bagging and random forest, TAGGING applied a natural model averaging weight to combine base learners and did not require a tuning process. Another direction of future work could be related to exploring sophisticated ensemble learning algorithms. Regularized linear regression on the base learners can be easily implemented (Friedman and Popescu 2008). Alternatively, meta-learning strategies such as stack regressions (Breiman 1996b) are approaches based upon the parallel training of multiple learning programs, followed by a meta-learning stage to stack them in a principled fashion.

The prediction ability of TAGGING models is not sufficient to outperform current standard GP methods, such as GBLUP. Our results suggest, however, that TAGGING can simultaneously match the prediction ability of the GBLUP model (with a bit of complementarity that can be exploited in additional ensembling) while also providing information on important genomic regions, which can be utilized in gene discovery. The improved prediction ability of the ensemble models over conventional QTL mapping imply they can better model the true QTL architecture, for example, by highlighting important genomic regions instead of relying on point estimates of QTL effects and by ameliorating collinearity in dense genetic maps.

### Oligogenic and polygenic model complementation

The high heritabilities (more than 85%, line mean based) of the three traits implied a great proportion of trait variation should be attributable to differences between maize lines after accounting for known environmental effects (Buckler *et al.* 2009; Kump *et al.* 2011; Peiffer *et al.* 2014). Traditional additive QTL models nevertheless provide only moderate prediction ability. This indicates that the genetic factors underlying these high heritable traits are complex or might not be approximated well without considering more complex model hypothesis. No strong specific digenic interaction was found in NAM populations for the studied traits, although it is still possible that polygenic additive by additive effects are important, even if we have not mapped specific interactions so far. Considering the epistasis may be one piece of the missing components, fitting feasible epistasis effects that can account for moderate or large effects seems practical in improving prediction accuracy, especially in our TAGGING framework where the hypothesis spaces can now be more easily searched. Previous studies showed that infinitesimal GP model (GBLUP or ridge regression models) outperformed QTL-based model in predicting complex traits for both multifamily populations (Peiffer *et al.* 2014) and biparental segregating populations (Lorenzana and Bernardo 2009; Guo *et al.* 2012). The opposite results were found for traits with major QTL (Zhao *et al.* 2014). In a plant breeding application, linear combinations of different GP (including Bayesian) models did not result in noticeable gain in GP accuracy (Heslot *et al.* 2012). Our results showed that the ensemble learners among well-tuned TAGGING-assisted QTL models and GBLUP models that come from the same genotypic information improved prediction, which suggests their useful complementation in prediction of complex traits. Furthermore, the factorial experiment of combining varied model predictions suggest that aggregating models that use different genotypic information is advantageous in GP in the tested NAM populations. Leveraging the complementary effects among model assumptions and/or genetic information provides one more possible solution to achieve a better model specification to approach the ideal heritable variation.

## 
